# Do Musicians with Perfect Pitch Have More Autism Traits than Musicians without Perfect Pitch? An Empirical Study

**DOI:** 10.1371/journal.pone.0037961

**Published:** 2012-05-30

**Authors:** Anders Dohn, Eduardo A. Garza-Villarreal, Pamela Heaton, Peter Vuust

**Affiliations:** 1 Center of Functionally Integrative Neuroscience (CFIN), University of Aarhus, Aarhus, Denmark; 2 The Royal Academy of Music, Aarhus/Aalborg, Denmark; 3 Department of Neurology, University Hospital, and Neuroscience Unit, Center for Research and Development in Health Sciences (CIDICS), Universidad Autonoma de Nuevo Leon, Nuevo Leon, Mexico; 4 Department of Psychology, Goldsmiths University of London, London, United Kingdom; George Mason University/Krasnow Institute for Advanced Study, United States of America

## Abstract

Perfect pitch, also known as absolute pitch (AP), refers to the rare ability to identify or produce a musical tone correctly without the benefit of an external reference. AP is often considered to reflect musical giftedness, but it has also been associated with certain disabilities due to increased prevalence of AP in individuals with sensory and developmental disorders. Here, we determine whether individual autistic traits are present in people with AP. We quantified subclinical levels of autism traits using the Autism-Spectrum Quotient (AQ) in three matched groups of subjects: 16 musicians with AP (APs), 18 musicians without AP (non-APs), and 16 non-musicians. In addition, we measured AP ability by a pitch identification test with sine wave tones and piano tones. We found a significantly higher degree of autism traits in APs than in non-APs and non-musicians, and autism scores were significantly correlated with pitch identification scores (*r* = .46, *p* = .003). However, our results showed that APs did not differ from non-APs on diagnostically crucial social and communicative domain scores and their total AQ scores were well below clinical thresholds for autism. Group differences emerged on the imagination and attention switching subscales of the AQ. Thus, whilst these findings do link AP with autism, they also show that AP ability is most strongly associated with personality traits that vary widely within the normal population.

## Introduction

Absolute pitch (AP) is the ability to identify the pitch of a musical tone or to produce a musical tone at a given pitch without the use of an external reference pitch [1], [2]. The estimated prevalence of AP is frequently reported to be around 1 per 10,000 [1], [3], [4], although a higher prevalence has been reported among East Asian populations [5], [6]. AP possessors are able to retain accurate information about an isolated pitch along the one-dimensional continuum of auditory frequency, and they are able to label that pitch within the context of the western chromatic scale [7]. It has been argued that the development of AP depends on musical exposure in a critical period in early childhood [5], [8]–[10] as well as on possible genetic contributions [3], [4], [6], [11], [12]. However, many musicians who begin training early in life and come from musical families do not develop AP, and relatively little is known about the traits and personality features associated with AP ability.

It has been suggested that AP ability may be associated to some extent with certain deficits, since there seems to be an increase in prevalence of AP among people with sensory and developmental disabilities. For example, AP is frequently reported in individuals with congenital blindness [13], [14], Williams syndrome [15], [16], and autism spectrum disorder (ASD) [17]–[21]. In one study, Heaton et al. [22] investigated AP in an intellectually-able adult with autism who had not experienced early musical training and observed statistically superior pitch naming in comparison with musically-trained typically-developing controls with AP. These results suggest that the genesis of AP may be different in ASD, and that pitch information is encoded with increased specificity in these individuals.

In the current version of the Diagnostic Statistical Manual (DSM-IV-TR) [23], autism is one of a cluster of disorders that are characterized by impaired social and communicative skills and co-occurring repetitive behaviors. Asperger syndrome is a subcategory that is characterized by the same core deficits as autism, but unlike autism, is not associated with delays in attaining early language and cognitive milestones. However, research suggests that at later developmental stages, individuals diagnosed with Asperger syndrome may show comparable levels of symptom severity as those who had experienced language and cognitive delays but were intellectually able (high-functioning autism) [24] In recognition of this and other failures to reliably differentiate between the different sub-categories detailed in DSM-IV-TR, it is proposed that the upcoming revision of the manual (DSM-V) will include a single spectrum disorder (ASD).

A notable feature of ASD is that unusual skills, as noted in the study by Heaton et al. [22] appear to be fairly common. Theoretical accounts of autism, for example, the Enhanced Perceptual Functioning theory [25], [26], propose that individuals with ASD show superior perceptual discrimination and, in addition, display an analytical cognitive style with increased pattern discrimination abilities. Interestingly, Chin’s [27] two-factor model of AP describes a genetic predisposition toward an “analytical cognitive style”, and this may account for increased levels of AP in autism.

Although ASD is considered to be a clearly defined neurodevelopmental disorder there is an increasing recognition that some of its defining characteristics can be observed at sub-clinical levels in the general population. Baron-Cohen et al. [28] have developed the Autism-spectrum Quotient (AQ) questionnaire to measure social skills, communication, imagination, attention to detail and attention switching in typical populations. The AQ questionnaire includes 50 items (i.e. personal statements) and participants are required to indicate whether or not the statements apply to them. For example, the statement “*I enjoy social chit-chat*” is included in the communication subscale question set, and “*I prefer to do things the same way over and over again*” is included in the attention switching subscale. The score ranges from 0 to 50, with higher scores indicating a higher prevalence of autistic traits. Baron-Cohen suggests that individuals who score above 32 points should be considered to have clinically significant levels of autistic traits [28]. Three of the five different AQ subscales measure what has come to be known as the “triad” of impairments characterizing autism. Hence, these questions probe social and communication skills and a tendency to repetitive behavior. The remaining subscales probe for characteristics of autism that have been identified in experimental studies. These include difficulties in imagination, difficulties in attention-switching, and exceptional attention to detail. Difficulties in attention switching are associated with poor cognitive flexibility and are consistent with work showing impaired executive functions in ASD [29]. Exceptional attention has been associated with special skills and may be associated with an analytical cognitive style detailed in the models of autism outlined by Mottron [26] and Baron-Cohen [30]. Interestingly, given questions about the prevalence of AP in autism, an analytical cognitive style is an important component in the model of AP proposed by Chin [27].

Studies using the AQ with typical adults have shown that natural science students have higher AQ than students from the social sciences and humanities, and that mathematicians have higher AQ than non-mathematician scientists [28]. These findings are consistent with previous studies showing an association between science/maths skills and autistic conditions [31] using other methods. Other studies using the AQ have shown that high AQ scorers are faster to complete the Embedded Figures (EF) test compared with low AQ scorers, independent of global IQ scores [32]. The EF task requires individuals to locate a simple form that is embedded in a larger visual display. It provides a measure of the individual’s field independence, defined as the ability to isolate details from their surrounding context. An early study by Shah and Frith [33] revealed superior EF task performance in children with autism, and these more recent findings, showing similarly superior performance in typical individuals with high AQ suggest similarities in cognitive style across these two groups. However, there is some evidence that abnormalities in perceptual processing are characteristics of individuals with high AQ scores. In a study by Gomot et al. [34] it has been observed that individuals with high AQ scores demonstrate superior auditory novelty detection, revealed by shorter reaction time, in a task requiring response to target stimuli in an oddball paradigm. They also displayed activation of an unusually widespread network of brain regions that are also observed in individuals with a formal diagnosis of autism [34].

Recently, a preliminary interview-based study carried out by Brown et al. [35] examined individual differences associated with the presence of AP in groups of classical trained musicians. The subjects were classified as being definitely socially eccentric, somewhat eccentric, or not eccentric on the basis of interviewer’s notes regarding subjects’ communication style and nonverbal behavior. The results showed that individuals classified as “socially eccentric” were more likely to be AP possessors. Whilst these findings are intriguing, it must be noted that the data were largely qualitative in terms of being interview-based and the groups were not matched for age, age of onset of musical training, or musical preference.

In the present study, we aimed to determine whether or not musicians with AP show increased levels of autism traits compared to matched groups of musicians without AP and non-musicians. Accordingly, we measured the autism traits of the participants quantitatively by administering the AQ and the level of AP ability using a pitch identification test, on the hypothesis of a correlation between the two. Finding higher scores on the AQ subscales measuring social and communication deficits in our AP group would be consistent with the results of Brown et al. [35], whereas differences on the subscales measuring attention-switching and attention to detail would support the model by Chin [27] who claim that AP is associated with detailed processing style. Finally, we examined whether musical abilities vary with degrees of autism traits and whether the level of AP ability is reliably related to musical aptitude.

## Methods

### Ethics Statement

This study was approved by the local ethics committee (De Videnskabsetiske Komiteer For Region Midtjylland, Denmark) and was performed in accordance with the Code of Ethics of the World Medical Association (Declaration of Helsinki). Written informed consent was obtained from each participant after detailed explanation of the experimental procedure and the object of the study.

### Participants

Fifty participants with a mean age of 27 years (*SD* = 6.5, range = 19–43) were recruited for the study. They consisted of the following: 16 musicians with self-reported AP (APs), 18 musicians without AP (non-APs), and 16 non-musicians. The three groups were matched with regard to gender (APs: 13 male, 3 female; non-APs: 13 male, 5 female; non-musicians: 13 male, 3 female, χ^2^ = .55, *p*>.05) and age (APs: *M* = 29.0, *SD* = 7.3; non-APs: *M* = 29.2, *SD* = 7.0; non-musicians: *M* = 23.6, *SD* = 2.8; *H*(3) = 5.7, *p*>.05). The two groups of musicians were also matched with regard to age of onset of musical training (APs: *M* = 5.5, *SD* = 2.1; non-APs: *M* = 5.6, *SD* = 2.0, *t* =  −0.08, *p*>.05) as well as to their preferred style of music (APs: 5 classical musicians, 11 jazz/rock/pop musicians; non-APs: 6 classical musicians, 12 jazz/rock/pop musicians, χ^2^ = .017, *p*>.05). All participants received compensation for being in the study.

### Pitch Identification Test

To confirm self-reported AP and to distinguish APs from non-APs, the two musician-groups were asked to complete an online pitch identification test (PIT) available from Athos et al. [36] and developed by Baharloo et al. [37]. The non-musicians were not asked to take this test since it requires familiarity to musical note names. The PIT consisted of 80 trials: 40 randomly-selected pure tones (i.e. computer-generated sine waves without overtones) and 40 randomly-selected digitized piano tones. The participants were asked to listen to the presented tones and to identify them by responding via an onscreen piano keyboard. A short practice test was given first to acquaint participants with the keyboard response on the computer screen and to adjust the volume of the computer.

The tones had duration of 1 second with a 2-second interlude between tone onsets. They were delivered in series of 10 tones, giving the participant an opportunity to pause between sets. Four of the pure tones and four of the piano tones were excluded from the scoring due to their position at the outermost range of the keyboard, resulting in 72 counting trials. Participants were given 1 point for each correct answer (maximum score for the pure tone test and the piano tone test together is 72) and 3/4 point for each error of a semitone. We averaged the participant’s scores in mean pure tones and mean piano tones, and those who scored above a threshold of 36 were designated APs, whereas the rest of the participants were designated non-APs. The probability of testing AP by chance alone with this threshold of 36 in total (pure tones + piano tones) is 1.21×10^−10^ [36]. Note that the mean expected score by chance is 14.25 with 95% of expected values lying between scores of 8.5 and 20.75.

### The Musical Ear Test

To examine the musical aptitude of the participants and to clearly distinguish musicians from non-musicians, all participants completed the musical ear test (MET), a newly developed test designed for measuring musical abilities objectively and quantitatively in both musicians and non-musicians [38]. The test consists of 104 trials in which participants listen to two musical phrases and subsequently judge whether or not they were identical by responding on an answer sheet. The first half of the test is a melodic subtest consisting of 52 pairs of melodic phrases, played with sampled piano sounds, and the other half is a rhythm subtest consisting of 52 trials with rhythmical phrases, played with wood block sound. Before each subtest, participants are given two example trials with feedback. Half of the trials in each session (26) are “same” trials and half are “different” trials, with the order randomized in both sessions. The participant’s score is the percentage of correct answers out of the 104 trials.

### Autism-spectrum Quotient

All participants also completed the Autism-spectrum quotient (AQ), a self-administered questionnaire developed by Baron-Cohen et al. [28] that quantitatively measures the degree to which an adult with normal intelligence has autistic traits. Although the scoring scale is not a diagnostic measure, those with autism spectrum disorder have been shown to score at the high end of the scale [28], [39], and it has been validated against clinical diagnoses [40]. The test consists of 50 items, made up of 10 questions assessing five subscales: “social skills”, “attention switch”, “attention to detail”, “communication” and “imagination”. Half the questions are formulated to elicit an ‘agree’ response and the other half a ‘disagree’ response.

The participants answered the questions on a computer by clicking their response on a multiple choice table with four response boxes entitled “definitely agree”, “slightly agree”, slightly disagree”, or “definitely disagree”. We used the scoring procedure described by Baron-Cohen et al. [28] ranging from 0 to 50, with higher scores indicating greater inclination towards autistic traits. Hence, higher scores in the subscales indicate poorer social skill, poorer attention switching, stronger attention to detail, poorer communication skill, and poorer imagination.

### Procedure

The APs were primarily identified through word of mouth and through advertisements at the Danish Royal Academy of Music and the Music Department at the local university. The non-APs and the non-musicians were found subsequently through advertisements and were selected randomly using matching criteria.

The participants were tested individually. First, the participant answered a short questionnaire regarding age, gender, musical background, and experience with AP. The non-APs and the non-musicians were told that they did not have to answer the questions regarding AP experience. Then, the APs and non-APs completed the PIT on a computer with in-ear-stereo headphones. Next, each participant completed the MET with paper and pen, with the audio part played back from the computer. Next, they completed the AQ on the same computer.

The APs were tested for 90 minutes using EEG and MRI as part of other ongoing experiments before the behavioral tests. No participants reported problems with either the auditory stimuli or the answering procedure in the PIT, the MET, and AQ.

### Statistical Analysis

Data from each group were assessed for normality with the Kolmogorov-Smirnov test and the Shapiro-Wilk test, which revealed violations of normality assumptions. Accordingly, we used non-parametric tests for subsequent statistical analyses.

We compared the AQ score between the three groups using the Kruskal-Wallis test. To investigate which autistic factors contribute most to the difference between groups, we studied the five factors of the AQ using Kruskal-Wallis test. This analysis was followed up by Mann-Whitney rank sum tests to investigate the differences between groups. Effect sizes were calculated as: 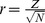 from the Mann-Whitney rank sum tests. Furthermore, we performed Spearman correlation to investigate the relation between the PIT score and the AQ score.

We used the Kruskal-Wallis test for comparison of age and for the total MET scores from all three groups, and Mann-Whitney rank sum test was used for comparison of PIT scores from the APs and non-APs. All analyses were corrected for multiple comparisons using the Bonferroni correction for p<.05.

## Results

### Autism-spectrum Quotient

**Figure 1:**
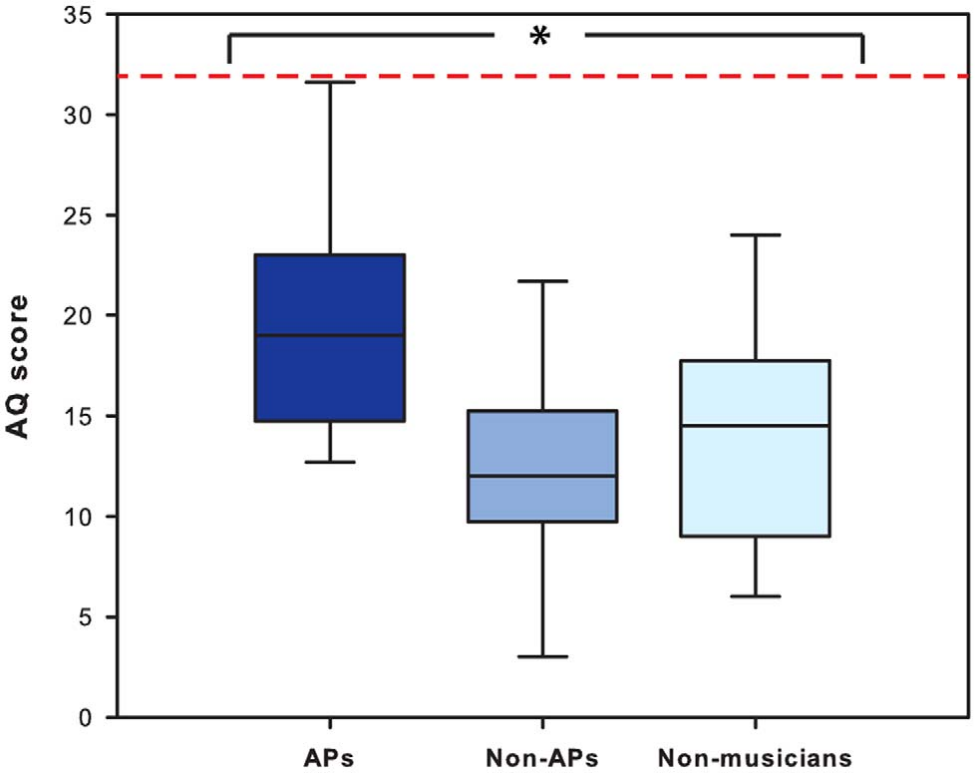
AQ score. Box plot showing the autism-spectrum quotient (AQ) score of absolute pitch possessors (APs), musicians without absolute pitch (non-APs), and non-musicians. The red dashed line shows the proposed AQ cut-off for distinguishing individuals who have clinically significant levels of autistic traits, according to Baron-Cohen (2001).

The mean AQ scores are shown in Table 1. We found a statistically significant difference between the three groups (*H*(3) = 11.32, *p* = .003). To follow up this finding, Mann-Whitney tests revealed a significant difference between APs and non-AP’s (*U* = 51.00, *p* = .007, *r* =  −.55) and between APs and non-musicians (*U* = 62.00, *p* = .036, *r* =  −.43). However, no significant difference was found between non-APs and non-musicians (*U* = 128.50, *p*>.05) (Fig. 1). These results show that absolute pitch possessors score significantly higher than individuals without absolute pitch in the autism-spectrum quotient.

**Table 1:** Descriptive statistics of AQ scores.

AQ scores	*Mean*	*SD*
APs	19.6	6.0
Non-APs	12.8	6.0
Non-musicians	13.8	5.9
AS/HFA	35.8	6.5

Means and standard deviations (*SD*) of autism-spectrum quotient (AQ) scores of musicians with absolute pitch (APs), musicians without absolute pitch (non-APs), non-musicians, and a group of adults with Asperger syndrome (AS) or high-functioning autism (HFA) as reported in Baron-Cohen [28].

#### Correlation

**Figure 2:**
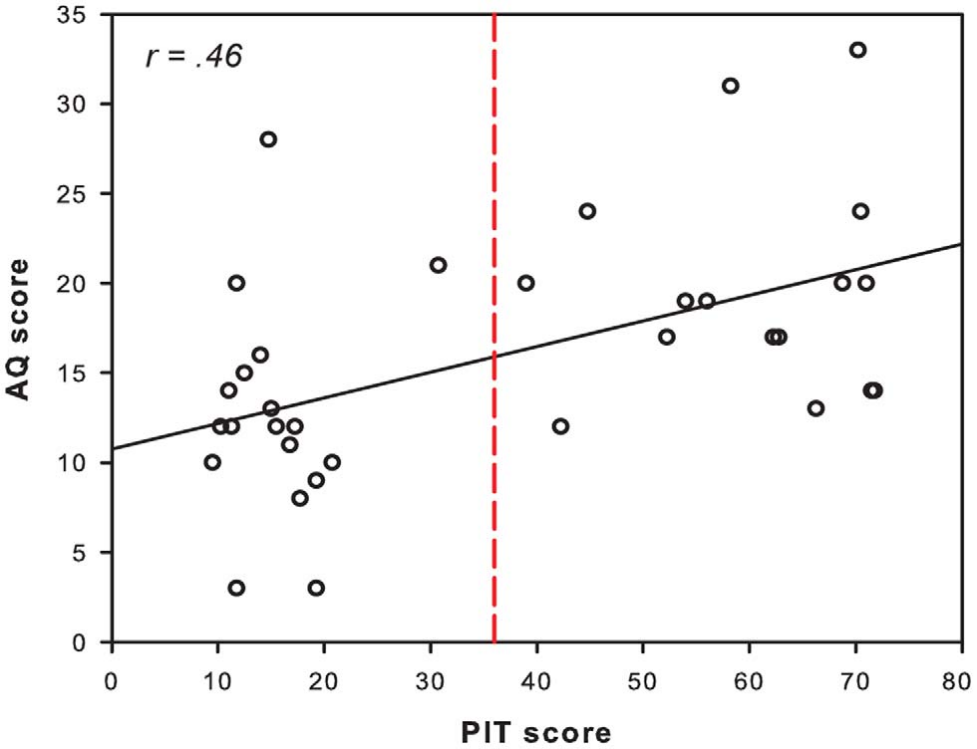
AQ/PIT scores. Scatterplot showing the autism-spectrum quotient (AQ) score as a function of the pitch identification score (PIT). The red line indicates the absolute pitch (AP) cut-off. To the right side of the red line are the musicians with AP (APs) and to the left side are the musicians without AP (non-APs).

We found a positive correlation between the PIT score and the AQ score (*r* = .46, *p* = .003) (Fig. 2). This shows that high scores among musicians on the pitch identification test are associated with high scores on the autism-spectrum quotient. There was no correlation between the MET and the AQ scores, suggesting that musical abilities may not vary with the level of autism traits.

### Autism-spectrum Quotient Factors

**Figure 3:**
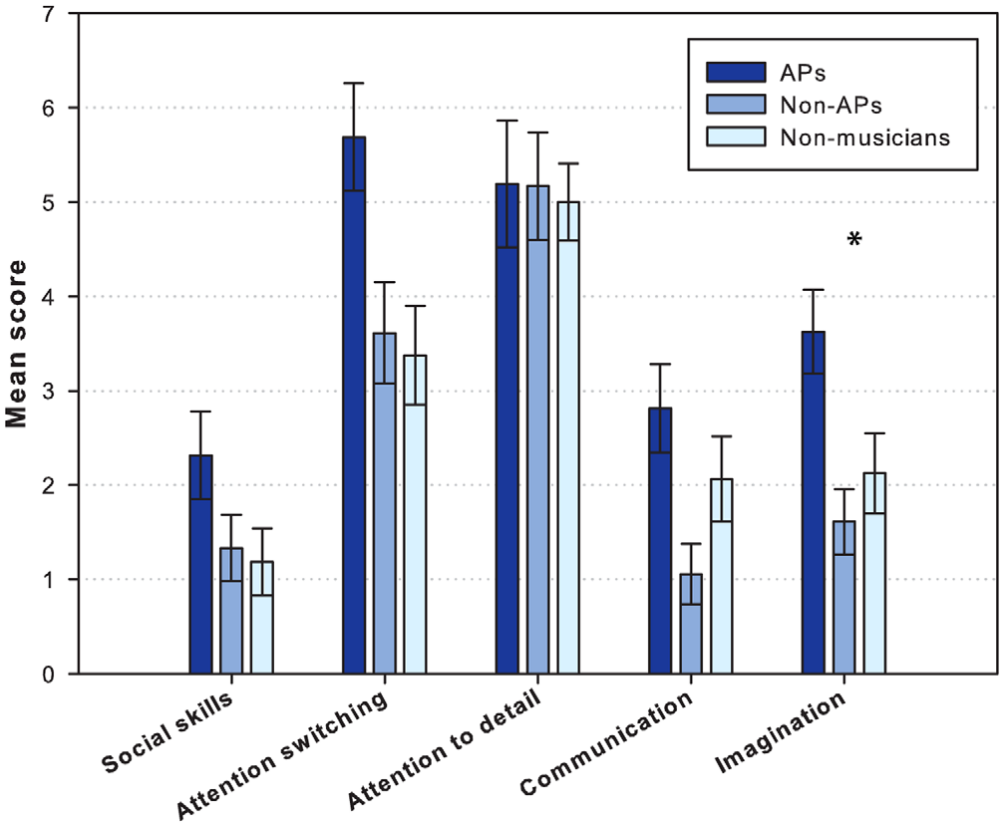
AQ factors. Bar plot showing the mean autism-spectrum quotient (AQ) factor score of all groups. The error bars indicate the standard error of the mean.

The descriptive data and statistical results are presented in Table 2 and Figure 3. We found a statistically significant difference in the “imagination” factor (*H*(3) = 10.2, *p* = .049); the APs showed significantly higher AQ score in imagination (i.e. less imaginative). This indicates that the imagination factor contributed strongly to the AQ score. However, we cannot rule out contributions from other factors, such as attention switching, because we noted also differences in that factor, although they failed to reach conventional levels of statistical significance.

**Table 2:** Statistical results of AQ factors.

	APs	Non-APs	Non-musicians	Kruskal-Wallis	
AQ factors	*Mean (SD)*	*Mean (SD)*	*Mean (SD)*	*H*	*p*
**Social skills**	2.3 (1.9)	1.3 (1.5)	1.2 (1.4)	4.1	Ns
**Attention switching**	5.7 (2.3)	3.6 (2.3)	3.4 (2.1)	9.1	(.08)
**Attention to detail**	5.2 (2.7)	5.2 (2.4)	5.0 (1.6)	0.1	Ns
**Communication**	2.8 (1.9)	1.1 (1.4)	2.1 (1.8)	8.1	Ns
**Imagination**	3.6 (1.8)	1.6 (1.5)	2.1 (1.8)	10.2	.049

Means and standard deviation (*SD*) of autism-spectrum quotient (AQ) factor score for musicians with absolute pitch (APs), musicians without absolute pitch (non-APs), and non-musicians with standard deviation (*SD*) in parentheses. The *H* value relates to a Kruskal-Wallis test between the three groups. Ns  =  not statistically significant.

### Pitch Identification Test

**Figure 4:**
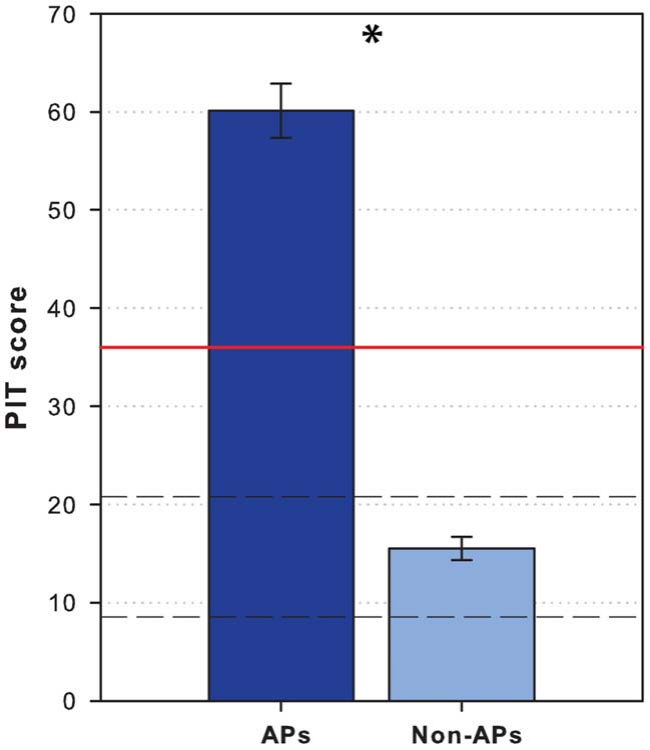
PIT score. Bar plot showing the mean pitch identification test (PIT) score of the absolute pitch possessors (APs) and the musicians without absolute pitch (non-APs). The error bars indicate the standard error of the mean. The red line indicates the threshold for possessing absolute pitch (>36), and the dashed lines indicate the range of scores expected by chance distribution, with a mean expected score by chance of 14.25, and with 95% of expected values lying between scores of 8.5 and 20.75.

The APs had a mean PIT score of 60.1 (*SD* = 11.1, ranging from 39 to 71.75), whereas the non-APs had a mean PIT score of 15.5 (*SD* = 5.1, ranging from 9.5 to 30.75) (Fig. 4). This shows that the APs are unambiguously distinguished from the non-APs (*U* <.0001, *p*<.0001) in that, they do not overlap.

### Musical Ear Test

**Figure 5:**
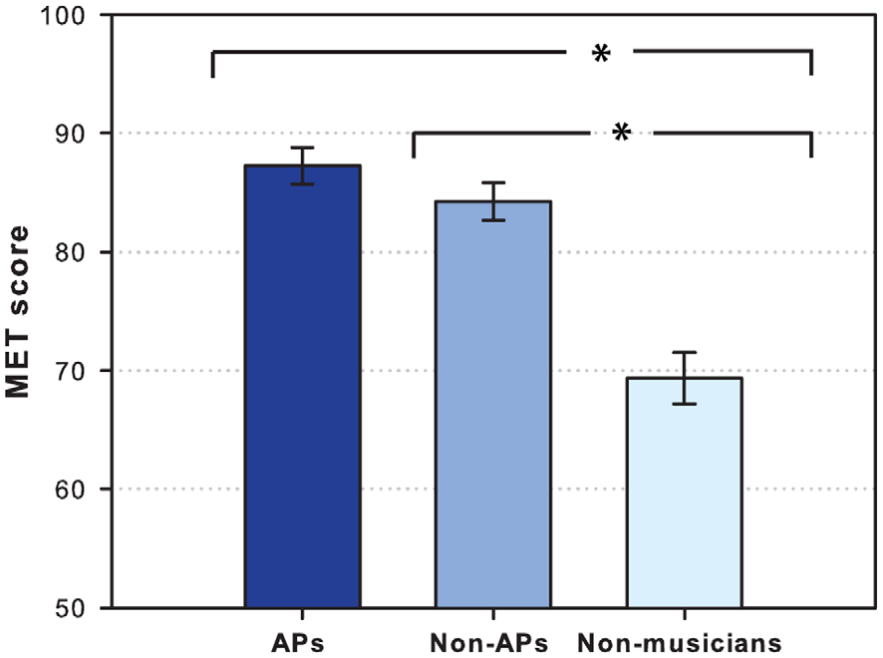
MET score. Bar plot showing the mean total musical ear test (MET) score of absolute pitch possessors (APs), musicians without absolute pitch (non-APs), and non-musicians. The error bars indicate the standard error of the mean.

The MET scores were distributed as follows: APs mean = 87.3% (*SD* = 6.1), non-APs mean = 84.2% (*SD* = 6.8), and non-musicians mean = 69.4% (*SD* = 8.6) (Fig. 5). A statistically significant difference was found between the three groups (*H*(3) = 26.23, *p*<.0001). Planned post-hoc Mann-Whitney tests revealed a significant difference between APs and non-musicians (*U* = 10.00, *p*<.0001) and between non-APs and non-musicians (*U* = 20.00, *p*<.0001). However, no statistically significant difference was found between APs and non-APs (*U* = 108.50, *p*>.05), and no significant correlation between PIT and MET was found (*r* = .24, *p*>.05).

These results suggest a clear distinction between musicians (with or without AP) and non-musicians, and that the level of AP ability among musicians is unrelated to the level of musical abilities as measured by the MET.

## Discussion

Here, we show that musicians with absolute pitch (AP) score higher than individuals without AP (musicians and non-musicians) on the autism-spectrum quotient (AQ), and that AP accuracy correlates with AQ. However, our results showed that the association between AQ traits and AP resulted from group differences in scores on the imagination and attention shifting subscales rather than on scores on the social and communication subscales.

This finding is surprising given that the results from Brown et al. [35] concluded that AP possessors (APs) are more likely to have impairments in social behavior than non-possessors (non-APs). Furthermore, they speculated that AP is an example of piecemeal information processing, an enhanced attention to isolated details of a configuration at the expense of attention to the whole. In the earliest study of AP in autism, Heaton, Hermelin & Pring [17] observed a highly significant correlation between pitch memory scores and scores from a cognitive task (block design) that was taken as a marker for a local bias or an analytical cognitive style characteristic of autism. However, later studies have failed to observe this association [18], [19], and it does not appear that a local processing style is a necessary precursor for AP in individuals with autism. Consistent with this conclusion are the results from the current study showing that APs and non-APs did not differ in their attention to detail. Hence, our findings do not indicate piecemeal information processing by musicians with AP as suggested by Brown et al [35], and may also challenge the importance of analytical style as outlined in the model of AP by Chin [27]. In one study of a musical savant with AP, Mottron et al. [41] noted features commonly observed in individuals with executive function deficits and suggested that absolute pitch may result from executive function difficulties, most notably a lack of cognitive flexibility, in a person with a marked interest for auditory stimuli. Whilst our comparison of AP and non-AP groups did not reveal a significant difference on the attention-switching factor, scores were markedly higher for the AP group, and that trend may add support to the suggestion that reduced cognitive flexibility is implicated in AP.

It is important to emphasize that even though our AP possessors achieved reliably higher AQ scores than our non-possessors, they did not, with one exception, have scores above 32 which is the cut-off for the DSM-IV-TR criteria for high functioning autism as suggested by Baron-Cohen et al. [28]. The only person in our study who exceeded that score was an AP possessor who obtained a score of 33 but did not evidence any social or communication disability and had never been given a diagnosis of autism or related disorder. Thus, our findings, whilst showing that AP possessors exhibit more traits associated with the broad autism phenotype than non-possessors and non-musicians, do not support the notion of increased social and communication disability in musicians with AP.

Interestingly, the musicians without AP had a lower mean AQ than the non-musicians (albeit not statistically significant). The results showing that the mean scores for the communication factor were twice as high for the non-musicians as for the musicians without AP are particularly striking. Whilst the sample size may not be large enough to allow for a statistically significant difference across groups, the result does suggest that musicians without AP show minimal AQ traits. However, musicians constantly communicate with sound to create their musical artwork, and communication within musical ensembles and with audiences is an essential element of playing music [42]–[44]. This may explain why musicians without AP display even fewer communication impairments than non-musicians as measured by the AQ.

An interesting finding, possibly related to communicative abilities in musicians, was the observed difference between groups on the imagination subscale of the AQ. This factor clearly contributed notably to the difference in total AQ scores across the three groups. However, this finding should not be interpreted as evidence that the APs have high deficits in imagination. When comparing the mean imagination score of our groups of APs, non-APs, and non-musicians (see Table 2) with the controls and student controls in Baron-Cohen’s AQ study [28] (Controls: *M* = 2.3, *SD* = 1.7; Students: *M* = 2.5, *SD* = 1.9*)*, it becomes clear that the score of the non-AP musicians in our study was extraordinarily low. This could be explained by the fact that musicians in general use their imagination in order to express a certain musical style with creativity and empathy, especially in music that involves improvisation. However, when we compare the imagination score of the APs in our study with Baron-Cohen’s group of adults with Asperger syndrome or high-functioning autism (*M* = 6.4, *SD* = 2.1), we see that the two groups differ markedly. Hence, we propose that while musicians with AP may be less imaginative than musicians without AP and non-musicians, they do not show clinically significant imagination deficits.

One limitation in the study of musicians with and without AP by Brown et al. [35] was that they did not include a group of non-musicians in their study. Our inclusion of a non-musician group enabled us to draw comparisons between musician and non-musician groups. Further, our use of the AQ enabled us to provide a more detailed account of the traits associated with AP.

In conclusion, our findings show that AP ability is not associated with deficits in social and communication abilities in typical populations and this challenges previous work making such links. Future research might focus on further exploring our key finding showing differences in imagination in APs and non-APs. Whilst the group difference in attention switching failed to reach statistical significance in our study, scores from this subscale contributed substantially to the observed difference in total AQ scores and it would be interesting to explore cognitive flexibility and its relationship with imagination in a larger group of individuals with AP. Results obtained by Mottron et al., suggested that reduced cognitive flexibility may be implicated in AP in autism, and extending this work to the non-autistic population may provide important new insights into absolute pitch ability.
